# The cubital tunnel syndrome caused by intraneural ganglion cyst of the ulnar nerve at the elbow: a case report

**DOI:** 10.1186/s12883-018-1229-7

**Published:** 2018-12-22

**Authors:** Pengfei Li, Danfeng Lou, Hui Lu

**Affiliations:** 1 Department of Plastic and Aesthetic Center, The First Affiliated Hospital, College of Medicine, Zhejiang University, #79 Qingchun Road, Hangzhou, Zhejiang Province 310003 People’s Republic of China; 2 Department of Infectious Diseases, Shulan(Hangzhou) Hospital, #848 Dongxin Road, Hangzhou, Zhejiang Province 310000 People’s Republic of China; 3 Department of Hand Surgery, The First Affiliated Hospital, College of Medicine, Zhejiang University, #79 Qingchun Road, Hangzhou, Zhejiang Province 310003 People’s Republic of China

**Keywords:** Cubital tunnel syndrome, Intraneural ganglion cyst, Ultrasound-guided aspiration

## Abstract

**Background:**

Cubital tunnel syndrome is common nerve compression syndrome among peripheral nerve compression diseases. However, the syndrome caused by intraneural ganglion cysts has been rarely reported. Medical approaches, like ultrasound-guided aspiration and open surgical treatment remain to be discussed.

**Case presentation:**

A 57-year-old woman presented with occasional pain, numbness and paralysis in her left hand and a palpable, painless mass in the ulnar side of her left elbow. Ultrasound-guided aspiration of the mass was performed to decompress the ulnar nerve. The patient experienced an evident release of pain in her hand, but symptoms of numbness and paralysis recurred 3 months later which greatly bothered the patient’s daily life. After evaluation, we had to perform an open surgery to excise the cyst. External neurolysis and anterior subcutaneous transposition were done. The patient was followed up for 2 years, and she made a complete recovery with no functional limitation.

**Conclusions:**

The symptoms caused by intraneural ganglion cyst can be alleviated by accurate puncture. But puncture may be not complete and symptoms could recur. Complete external neurolysis can be counted as a complete and reliable treatment. Therefore, early diagnosis, careful preoperative imaging assessment and full decompression can be expected to receive a good rehabilitation.

## Background

Cubital tunnel syndrome is known as the second most common upper extremity compressive neuropathy, which may cause chronic ulnar nerve dysfunction, like permanent loss of sensation, muscle weakness, and joint contractures [1, 2]. However, cubital tunnel syndrome caused by intraneural ganglion cysts has been rarely reported [3–6]. Their pathogenesis has been controversial. Different treatments have been recommended [7, 8]. We reported a case of cubital tunnel syndrome caused by intraneural ganglion cyst of the ulnar nerve at the elbow. We first tried aspiration of the cyst. But there was a recurrence 3 months later. We then fully excised the cyst and externally relaxed the ulnar nerve which gave the patient a complete recovery. The advantages and disadvantages of these two treatments were discussed.

## Case presentation

**Fig. 1:**
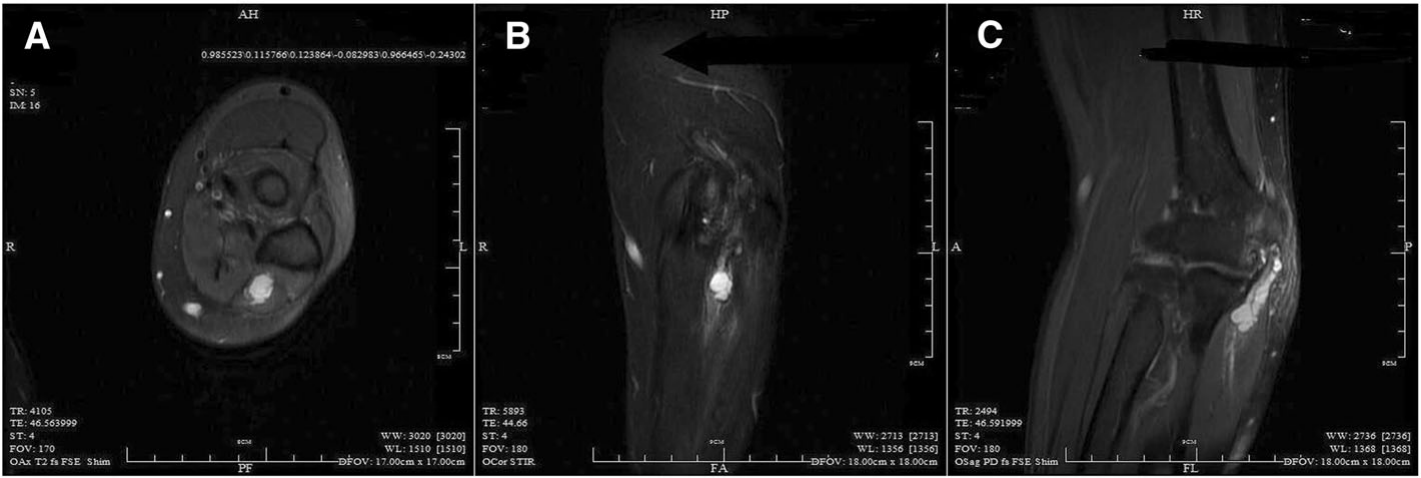
Display of magnetic resonance imaging before medical intervention. **a** Hyperintense lesion in ulnar nerve at cubital tunnel on T2-weighted image in transverse section. **b** Hyperintense lesion in ulnar nerve at cubital tunnel on T2-weighted image in coronal section. **c** Hyperintense beaded lesion in ulnar nerve at distal elbow on T2-weighted image in median sagittal section

A 57-year-old female patient presented in our clinic with complaints of occasional pain, numbness and paralysis in her left hand and a palpable, painless mass in the ulnar side of her left elbow for the last 2 months. There was no history of trauma. Besides the discomfort in the left elbow, the patient had a history of lumbar disc protrusion and hypertension, which was well controlled with medication. No other medical related history could be traced. Physical examination showed a painless mass (about 1 cm*2 cm) in the ulnar side of her left elbow with no inflammation. Neurologic examination revealed light numbness on the ulnar side of her left hand and fingers. No pathological sign was detected positive. Electromyography (EMG) showed the ulnar nerve across the elbow was injured. Magnetic resonance imaging (MRI) disclosed a subcutaneous irregular abnormal signal in the upper ulnar side of left forearm, hyperintense on T1and T2-weighted image which was considered to be a benign lesion, and joint effusion in the left elbow (Fig. 1). X-ray showed degenerated change in the left elbow joint. Laboratory studies revealed the routine blood test, tumor markers, erythrocyte sedimentation rate (ESR), and high-sensitivity C-reactive protein were all within normal range. The mass was considered to be a cystic form disease which compressed the ulnar nerve. With the guidance of ultrasound, we first located the cyst. Precise puncture and aspiration were made with a 18G biopsy needle (Gallini, Italy) to evacuate mucinous material inside the cyst. The mass deflated mostly and the patient experienced an evident release of the pain with no significant improvement in other symptoms.

**Fig. 2:**
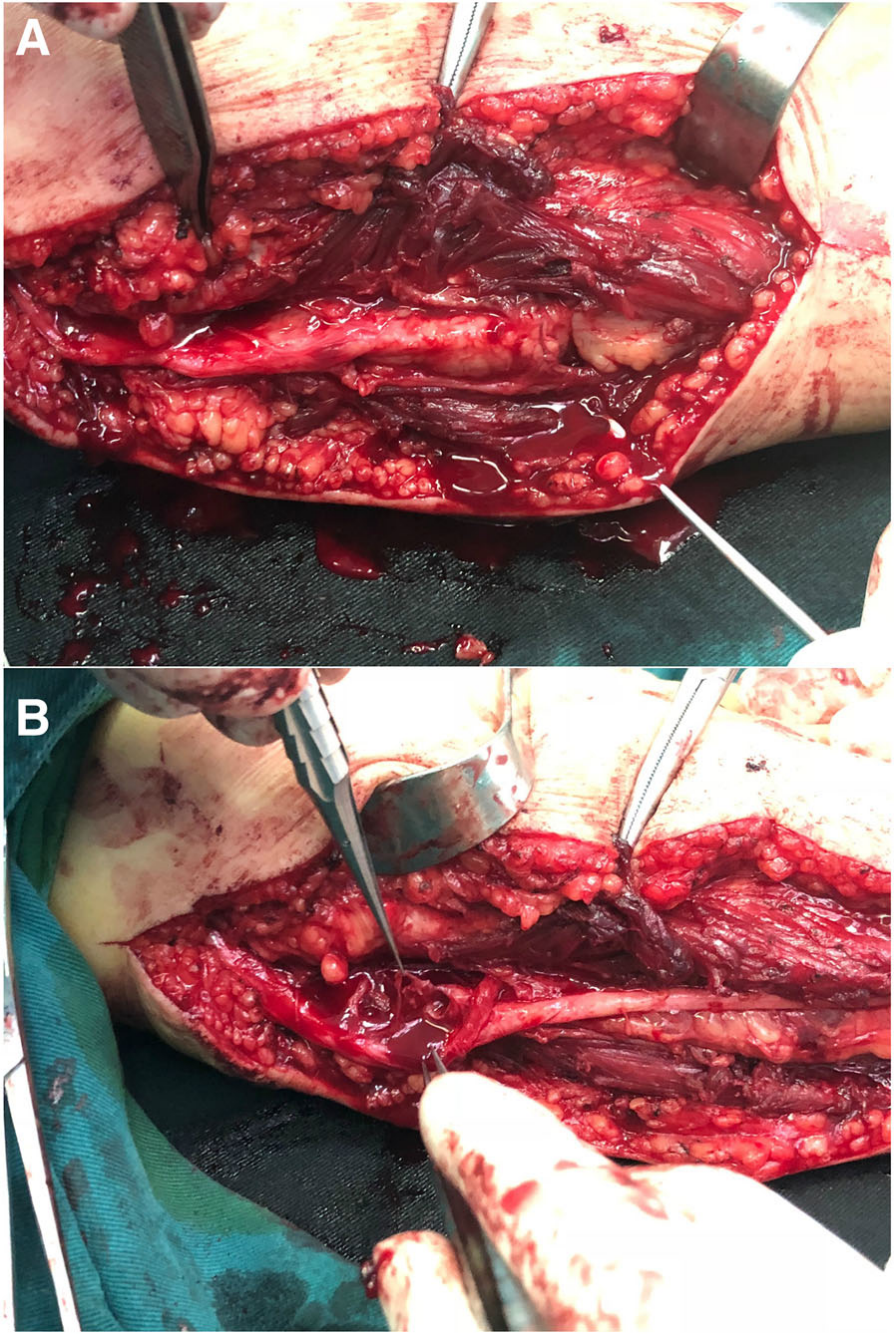
Intraoperative findings: **a** Initial exposure revealed thickened epineurium of the ulnar nerve in the elbow and compression of fat and vascular tissue in the distal side of the ulnar nerve. **b** Further dissection disclosed ruptured cystic wall within nerve fibers

Three months later, the patient came to the clinic complaining the recurrence of symptoms of numbness and paralysis which still tremendously affected her daily life. Further evaluation indicated that open surgery was inevitable. The ulnar nerve was then surgically explored along its trajectory with a curve incision. The ulnar epineurium at cubital tunnel was thickened and the tunnel was constricted. After careful dissection, ruptured cystic wall was disclosed within nerve fibers (Fig. 2). Full excision of the cystic wall was performed and a sample of the lesion was sent for frozen section. Distal constriction of the ulnar nerve by fat and vascular tissue was discovered and complete decompression was operated. External neurolysis of the ulnar nerve was carefully done together with anterior subcutaneous transposition to relocate ulnar nerve on the soft tissue bed.

**Fig. 3:**
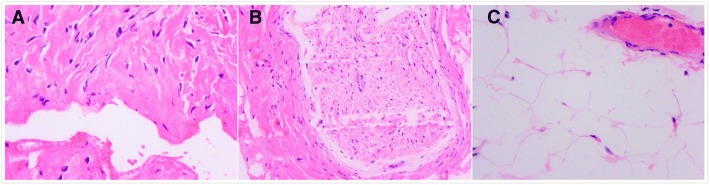
Histopathology of the lesion demonstrating features of an intraneural ganglion cyst. **a** Fibrous tissues of cystiform (40*10 H&E). **b** Nerve tissue (20*10 H&E). **c** Fat and vascular tissue in the distal of the ulnar nerve (40*10 H&E)

Histopathology revealed that the sample was fiber tissues in cystic wall (Fig. 3). Reporting diagnosis was intraneural ganglion cyst. The patient was evaluated 2 weeks after the surgery with improvement in motor function and some minor alleviation in dysesthesia. Follow-up of 2 years showed complete release in the symptoms and the latest MRI imaging revealed no sign of recurrence.

## Discussion and conclusions

Cubital tunnel syndrome is a commonly seen neuropathy of the upper extremity caused by entrapment of the ulnar nerve in the elbow [1, 2]. One rare cause of the syndrome is intraneural ganglion cysts which are benign, mucinous, non-neoplastic lesions of the peripheral nerves [3–5]. Mild to severe symptoms, ranging from discomfort, numbness, pain to disability, loss of function in the affected hand, can be felt in afflicted patients due to nerve compression [1, 9, 10]. The pathogenesis of intraneural ganglion cysts remains unclear. Though trauma was proposed to be the possible reason for the lesion [11, 12], the theory of articular unification which was proved by evidences was mainly accepted [3]. In our case, the latter was considered to be the cause.

Decompression of the enlarged nerves and resection of the articular branch are the pertinent treatments for intraneural ganglion cysts [3, 13]. Choice of interventions is generally based on severity of nerve compression, surgeons’ preference, and patients’ specific situations [14, 15]. Though there are a wide range of treatments to choose from, they do not allow the same results. Desy [13] et al. showed an increased rate of recurrence after primary surgery in his literature analysis. Thus, careful evaluation and cautious selection of treatment are of significance.

In this case, the patient suffered moderate pain, numbness and paralysis in her left hand which is applicable for ultrasound-guide diagnosis puncture [8, 16]. We tried ultrasound-guided aspiration first which achieved an obvious alleviation of the pain in the hand and deflated the palpable mass about the elbow. With a minimal invasion, the aspiration could deflate the cyst to decompress the constriction allowing reinnervation and recovery. While guided by ultrasound, it is highly possible that the aspiration would injure the nerve which was adjacent to the cyst. Although no sample could be collected for pathological examination, the cyst was initially considered to be intraneural, instead of extraneural. In addition, the numbness and paralysis in her left hand remained 3 months later which indicated that there were still some compressions unhandled inside. Combined with the discovery from the open surgery, there were two problems retained. First, while the cyst was released, the epineurium was waiting to be decompressed. Second, complete dissection couldn’t be done to discover the distal entrapment by fat and vascular tissue of the ulnar nerve which was barely reported before.

The trajectory of the ulnar nerve was thoroughly explored surgically. Besides full excision of the cystic wall, another compression in the distal side of the ulnar nerve was relaxed. The patient experienced a full rehabilitation afterwards with no recurrence in the 2 years of follow-up.

From this case, it is indicated that there might be more anatomic factors that caused compression along the ulnar nerve which resulted in the cubital tunnel syndrome. Simply deflating the intraneural ganglion cyst could relieve the symptoms, but complete decompression demands for open surgery and external neurolysis.

The cubital tunnel syndrome caused by intraneural ganglion cyst needs to be treated seriously.

Aspiration may be not complete and symptoms could recur. Complete external neurolysis in open surgeries is a more effective and reliable method than ultrasound-guided aspiration. Early diagnosis, careful preoperative imaging assessment and full decompression can be expected to achieve a good rehabilitation.

## Abbreviations

EMG: Electromyography
ESR: Erythrocyte sedimentation rate
MRI: Magnetic resonance imaging
